# Role models in a preventive program for hand eczema among healthcare workers: a qualitative exploration of their main tasks and associated barriers and facilitators

**DOI:** 10.1186/s12895-015-0033-y

**Published:** 2015-08-20

**Authors:** Anne M Bruinewoud, Esther WC van der Meer, Joost WJ van der Gulden, Johannes R Anema, Cécile RL Boot

**Affiliations:** Department of Public and Occupational Health, EMGO Institute for Health and Care Research, VU University Medical Center, Van der Boechorststraat 7, 1081 BT Amsterdam, The Netherlands; Department of Primary and Community Care, Centre for Family Medicine, Geriatric Care and Public Health, Radboud University Nijmegen Medical Centre, PO Box 9101, 6500 HB Nijmegen, The Netherlands; Body@Work, Research Center Physical Activity, Work and Health, TNO-VU University Medical Center, Amsterdam, The Netherlands; Research Center for Insurance Medicine AMC-UWV-VU University Medical Center, Amsterdam, The Netherlands

**Keywords:** Role models, Healthcare workers, Hand eczema, Implementation, Facilitators, Barriers

## Abstract

**Background:**

Role models often play a role when implementing guidelines in healthcare. However, little is known about how role models perform their respective roles, or about which factors may hamper or enhance their functioning. The aim of the present study was therefore to investigate how role models perform there role as a part of a multifaceted implementation strategy on the prevention of hand eczema, and to identify barriers and facilitators for the performing of their role.

**Methods:**

The role models were selected to become a role model and received a role model training. All role models worked at a hospital. In total, 19 role models, were interviewed. A topic list was used focussing on how the role models performed their role and what they experienced to be facilitators and barriers for their role. After coding the interviews, the codes were divided into themes.

**Results:**

This study shows that the main tasks perceived by the role models were to raise awareness, to transfer information, to interact with colleagues about hand eczema, to provide material, and to perform coordinating tasks. Barriers and facilitators were whether the role suited the participant, affinity with the topic, and risk perception.

**Conclusions:**

Most role models performed only the tasks they learned during their training. They mentioned a wide range of barriers and facilitators for the performing of their role. To enhance the functioning of the role models, a suggestion would be to select role models by taking into account prior coaching experience.

**Trial registration:**

Trial registration number: NTR2812

## Background

When implementing guidelines in hospitals, multifaceted implementation strategies have shown to be more effective than single strategies [1]. As recent studies have showed that participatory programmes have been tested successfully in healthcare employees [2, 3], role models often play an important role in interventions. In 2010, Ploeg et al. [4] found that role models can influence the use of guidelines by dissemination of information, by being persuasive practice leaders and by tailoring the guideline implementation strategies to an organizational context. Although the experiences of role models in interventions on occupational skin diseases have been studied in wet work settings [5], it is still unknown which factors may hamper and/or enhance the role model’s function in a healthcare setting. To improve interventions that aim to increase adherence to guidelines among hospital staff, deeper insight into these factors is crucial.

Role models are often part of interventions that aim to manage changes in behaviour or to implement guidelines in healthcare [6, 7]. Role models are seen as one of the most important factors for the successful implementation of recommendations that aim to change workers’ behaviour [8] as they influence the subjective norm [9]. According to the Theory of Planned Behaviour [10], the subjective norm is one of three intermediate variables that form the intention to perform desired behaviour [11].

Changing the behaviour of workers within a healthcare setting is difficult, as is demonstrated by the low compliance to guidelines in hospitals [12]. Erasmus et al. [13] concluded that within the field of hand hygiene, a lack of positive role models among hospital staff during daily practice may hinder compliance to the proposed recommendations. This was explained by the finding that participants copy the behaviour of their superiors. Based on the research of Erasmus et al. [13] we hypothesized that the use of role models can positively contribute to the prevention of occupational hand eczema among healthcare workers.

Healthcare workers like nurses are at increased risk for developing hand eczema [14, 15] as occupational exposure to irritants like water greatly increases the likeliness for developing this skin disease [16]. Prevention is necessary, because recent research showed that the prevalence of hand eczema in healthcare workers is double the prevalance in other populations [17] and that the skin disease is one of the most prevalent work-related diseases in Europe [18].

To reduce hand eczema among healthcare workers, a multifaceted implementation strategy was developed in which healthcare workers were trained as role models as a part of this strategy. Previously, role models were often chosen based on their position in the organisation or department [8, 19]. In the present study role models were allocated alternatively, based on their representativeness, their influence on colleagues, and their motivation.

The aim of this study was to *qualitatively* explore what ‘placed’ role models considered to be their main tasks, and to gain insight into facilitators and barriers that could have influenced their role as a role model.

## Methods

### Design and participants

The participants of this study participated in the intervention group of the Hands4U study who received a multifaceted implementation strategy. The Hands4U study is a two-armed clustered randomized controlled trial. The goal of this study was to evaluatie the (cost-) effectiveness of the multifaceted implementation strategy and to investigate barriers and facilitators that arise in the use of this strategy. In total, 1649 Dutch healthcare workers participated in the trial of which 876 participated in the intervention group. More details on the Hands4U study have been published elsewhere [20]. Within the multifaceted implementation strategy used in this study, evidence-based recommendations were given about preventive measures for hand eczema [21]. The strategy contained five parts: 1) education; 2) participatory working groups who identified problems with adherence to the recommendations, found solutions for these problems, and implemented these solutions within their department; 3) role models who helped and encouraged their colleagues to increase adherence to the recommendations; 4) reminders (posters); and 5) a leaflet containing the recommendations.

All role models were members of the participatory working groups who followed an hour and a half educational session about hand eczema. The training consisted of a lecture by an occupational nurse and a role play. Topics were: dealing with resistance from colleagues, motivational interviewing, and principles of the stages of change model. Role models were trained on how to promote and enhance the implementation of recommendations for the prevention of hand eczema within their department, and on how to be a role model for their colleagues.

A total number of 70 role models participated in the Hands4U study, working at 23 departments. For the interview recruitment of the role models for the present study, convenience sampling was used. Role models were invited in two ways: 1) by asking them to participate during the last working group meeting, and 2) by sending them an e-mail with the request to participate. Variety in participants was desired, and was based on the following characteristics: the hospital and the department where the participants worked, gender, having patient contact or not, and having an executive function or not. In total, 19 role models from 14 different departments were interviewed. At the time the interviews took place, all participants have been performing their role for a period longer than six months.

### Data collection and ethical considerations

The interviews were conducted from December 2011 to January 2013 by EWCM. Before the interview, participants received an informative letter stating that the interview would take approximately 45 minutes. A semi-structured interview guide was used. The interviews were recorded and transcribed into Word-files. The interview covered two topics: 1) barriers and facilitators for the implementation of recommendations for hand eczema, and 2) experiences as a role model, and barriers and facilitators related to this role. The present study focused solely on the second topic. During the interviews, the participants were asked to explain what they considered to be tasks as a role model within their department, and what they perceived to be barriers and facilitators in performing their role as a role model. At the end of the interview, participants were asked whether all important topics were covered and if they wanted to add something to the conversation.

The Medical Ethics Committee of the VU University Medical Center approved this study. Most interviews took place within the participant’s department, in a private conference room. Due to the nature of the study, the ethics committee decided that no informed consent was needed.

### Data analysis

All interviews were read and quotes about the role model’s tasks, facilitators and barriers were identified by AMB, based on the method used in the study of Hooftman et al. [22]. As it is highly important to analyze the data systematically in qualitative research, the analyses were conducted by AMB in close colaboration with the second researcher (ECWM). AMB was not directly involved in the interviewing. The constant comparison method was used for the analyses, in which each item was compared with the rest of the data in order to establish analytical categories [23]. Facilitators were defined as factors that participants perceived as making it easier to perform their role as a role model. The barriers were factors that participants perceived as making it more difficult to perform their role as a role model within their department. First, five interview transcripts were randomly selected and read by AMB. Then, the interview transcripts were open-coded by AMB to identify relevant themes. To ensure that all the themes were identified, the interview transcripts were read and reread several times. Similar themes were clustered into categories and inter-subjective agreement was tested by a second researcher (EWCM) based on the first five interviews, as well as in one other interview that was randomly selected. When there was disagreement, the quote was discussed and afterwards a decision was made about the inclusion or exclusion of the quote. During consensus meetings, the third researcher (CRLB) solved disagreements on the coding and gave advice. The remaining interviews were read and efforts were made to detect quotes of the identified themes and, if applicable, to identify new themes. After analyzing the remaining interviews, we updated the themes and quotes were transcribed into codes categorized by main tasks, facilitators and barriers. Related segments and subthemes were subcategorized by AMB and seperately checked by EWCM and CRLB. Next, all codes were categorized into themes by three researchers (AMB, EWCM and CRLB). All quotes were translated from Dutch into English. There was no software package used to code the data other than Microsoft Word.

## Results

Of the 19 role models: 15 were women and four were men. Twelve of the participants worked in a department where there was patient contact (i.e. chirurgical units, intensive care units, dentistry), three worked in a kitchen, two worked in a laboratory, and two worked at the hospital’s pharmacy. The participants worked at different cities throughout the Netherlands: 13 in Groningen, and the remaining six worked in Nijmegen, Amsterdam, or Stadskanaal. Six participants had an executive function. Characteristics and quotes of the participants are shown in Table 1. In the following text quote numbers from Table 1 are given in square brackets.

**Table 1:** Characteristics and quotes of participants

Quote	Participant number, gender	
	Department (with/no patient contact)	
	Function (executive /not executive)	
1	6, Female	*‘When you see something fading away, you notice that with many things*, *you will have to repeat it*.’
	With patient contact	
	Executive function	
2	1, Male	‘*I am not the kind of person that says*: *“You have to do this or you have to do that” and that’s not the kind of person I want to be. And obviously, I do not have that role at the department… So not top down or in a compulsory way, but more like: “Did you know that hand eczema is often not visible, you might have it without knowing it?”’*
	No patient contact	
	Not executive function	
3	13, Female	‘*If you are trying to convince people of the importance, but you do not practice what you preach, they will never listen to you. So I think that’s the most important. That they see you do the things you say you do.’*
	With patient contact	
	Not executive function	
4	5, Female	*‘If I see something, I address people of course. And I say: “He guys, here’s the moisturizer, let’s use it.”’*
	With patient contact	
	Executive function	
5	18, Female	*‘Yes, and people ask: “Has the cotton under gloves already been ordered? What is the order status?” Because, of course, you told them that it was going to happen, they know it.’*
	No patient contact	
	Executive function	
6	8, Female	*‘Well, that role [the role of the role model] was performed more frequently by W. and P. and I performed the more coordinating tasks.’*
	No patient contact	
	Executive function	
7	1, Male	*‘Personally, I would have liked to know more about hand eczema and maybe the consequences of hand eczema and things like that. Then I would have been able to tell more about it, than I could after the first meeting.’*
	No patient contact	
	Not executive function	
8	10, Female	*‘Yes, and also personally I think it is important, because you still want to continue work for many years, so you do not want to have that kind of illness.’*
	With patient contact	
	Not executive function	
9	1, Male	‘*What made it easier was that I followed other courses previously, for instance ergonomy training, and first aid, and then you already know what is to be expected from you as a coach.’*
	No patient contact	
	Not executive function	
10	18, Female	*‘That’s because I am in a position where it is perhaps easier for me to address people than for an analyst in the laboratory. That might be the case.’*
	No patient contact	
	Executive function	
11	18, Female	‘*J. just arrived, she had just finished her studies and she has just started to work here, and she was also in the working group… I think she was trying to find her own way. She does not have the experience yet to address people or to arrange these kind of things.’*
	No patient contact	
	Executive function	
12	2, Female	‘*We all learned from it. So that’s positive as well. If you are going to do something and you think: “Yes, I already know that”, then you will not benefit from it. Yes, we all found it quite interesting and fun. Yes, that’s an advantage as well.’*
	No patient contact	
	Not executive function	
13	14, Female	*‘Well, the manager was also a member of the working group. That makes a difference when you wish to arrange things. Yes, that made it easier.’*
	With patient contact	
	Not executive function	
14	15, Male	*‘When one of them is suddenly entitled as ‘coach’, the other one says: ‘Why you?’ It is like a henhouse here.’*
	With patient contact	
	Executive function	
15	17, Female	*‘We work at very different locations, we do not have coffee breaks together, so it was difficult how I could announce this to everyone.’*
	With patient contact	
	Not executive function	
16	10, Female	*‘When you are working here, it is pretty busy. So it is not always easy to keep it in sight and that makes it a bit difficult.’*
	With patient contact	
	Not executive function	
17	9, Female	*‘But then of course, at a certain moment it is up to them to do something about it. You cannot force them, of course, that’s not always possible. That’s sometimes difficult.’*
	With patient contact	
	Not executive function	
18	10, Female	*‘We could have used that one colleague as an example, because it was very clearly visible and then you notice that people think: “Well, that’s not something I want, so I will do of course my best to avoid it.”*
	With patient contact	
	Not executive function	
19	16, Female	*‘”No, I don’t have hand eczema,” she said. So you can talk and talk, that didn’t help a thing. Or let me put it this way: it did not help here. So I consider that effect as very minimal.’*
	No patient contact	
	Not executive function	
20	7, Female	*‘It was sometimes really nice to be with the two of us, because when one of us was silent, the other one could come up with other nice arguments.’*
	With patient contact	
	Not executive function	
21	10, Female	*‘So that way you can show people examples by using for instance a leaflet… That makes it of course easier to say something or to show people that it can have a result.’*
	With patient contact	
	Not executive function	
22	14, Female	*‘Because it is a small and compact department, it has been easy to reach everybody.’*
	With patient contact	
	Not executive function	
23	12, Female	*‘Because it is not a major issue, I think, it has been given a relatively high priority, relative to the problem perception, and that can be difficult.’*
	With patient contact	
	Not executive function	

### Main tasks of the role models

There were five specific aspects that participants mentioned as their main tasks as a role model:1) to raise awareness and maintain mindfulness in regard to hand eczema; 2) to present information and modeling; 3) to have interaction with colleagues about hand eczema; 4) to provide relevant material; and 5) to perform coordinating tasks for the Hands4U project as a whole.

#### To raise awareness and maintain mindfulness on hand eczema

There were several ways in which role models tried to raise awareness for the topic of ‘hand eczema’: by periodically repeating relevant information to colleagues within their department, by directing attention to the theme, by informing new colleagues, and by showing enthusiasm and commitment to the topic. In many cases, the role models agreed with their manager about repeating the information on hand eczema regularly during meetings in order to keep focus on the topic [Table 1, Quote 1]. Furthermore, one participant stated that the enthusiasm of the role models was a determining factor for the implementation of all recomendations.

#### To present information and modeling

There were three main ways in which the role models provided information. First, transfer of knowledge was seen as a major task. Participants informed their colleagues about the benefits of using moisturizer and disinfectant and how to prevent hand eczema. In addition, participants noticed that it was important that their tone was not demanding because that did not fit with their personality or their role at the department [Table 1, Quote 2]. To be a good example for their colleagues was found to be another task of the role models, as they believed this was needed to convince their colleagues [Table 1, Quote 3]. Letting colleagues know that they have been assigned as a role model was named as a third way of information provision, for instance via a whiteboard announcement in the coffee room, by e-mail, and/or in team meetings.

#### Interaction with colleagues about hand eczema

The first aspect mentioned by participants was starting conversations with colleagues within the theme of ‘hand eczema’. This was often done when ‘unhealthy hand behaviour’ like not using moisturizer was noticed by the role model [Table 1, Quote 4]. A second aspect was answering questions from colleagues about hand eczema and the preventive measures associated with it. A participant explained [Table 1, Quote 5]: *‘Yes, and people ask: “Has the cotton under gloves already been ordered? What is the order status?” Because, of course, you told them that it was going to happen, they know it’.*

#### Providing material

Ensuring the availability of essential products for the prevention of hand eczema was considered to be very important by the role models. Providing material like moisturizers and gloves was therefore seen as one of their main tasks.

#### Coordinating tasks

Being a coordinator for the whole Hands4U project was the last task the role models mentioned. However, this task was mainly mentioned by participants in management positions [Table 1, Quote 6]. It seems that when there were several role models within a department, including a manager, there was a difference in the tasks that they performed.

### Facilitators and barriers

Several facilitators and barriers for performing their role were mentioned by the role models (Table 2). These factors were divided into *internal factors* (personal factors) and *external factors* (department factors or factors originating from colleagues or work situations). Some factors that were mentioned as a facilitator were also mentioned as a barrier in opposite direction (e.g. social support by colleagues was considered a facilitator, whereas lack of social support was mentioned as a barrier).

**Table 2:** Facilitators and barriers

Theme’s		Barriers	Facilitators
Internal	Knowledge about hand eczema	X	X
	Affinity with the topic	X	X
	Whether the role suited	X	X
	Attitude towards the role		X
External	Support or resistance colleagues	X	X
	Contact role model and colleagues	X	X
	Risk perception	X	X
	Amount of role models	X	X
	Availability of material		X
	Education		X
	Role suited the department		X
	Priority of hand eczema	X	
	Low risk at department	X	
	Little response of colleagues	X	
	Communication role models	X	

### Internal factors

#### Knowledge about hand eczema

Sufficient knowledge ensured that the role model felt more confident in his/her role and that he/she could come up with good arguments to motivate colleagues to alter their behaviour. Participants who indicated that they had sufficient knowledge about the prevention of hand eczema, reported that they had a higher self-efficacy in sharing this knowledge with their colleagues. When the role model lacked knowledge about (the prevention of) hand eczema, this was experienced as a barrier. One participant explained [Table 1, Quote 7]: *‘Personally, I would have liked to know more about hand eczema and maybe the consequences of hand eczema and things like that. Then I would have been able to tell more about it, than I could after the first meeting.’*

#### Affinity with the topic ‘hand eczema’

Acknowledging the need for the prevention of hand eczema as this might be a barrier to continue working was seen as an internal facilitator [Table 1, Quote 8]. On the other hand, a lack of affinity with the topic of ‘hand eczema’ was considered to be a barrier for performing tasks as a role model. Some participants mentioned that they found it hard to give priority to and stay enthusiastic about a subject that they would not mark as highly important outside of the research project.

#### Whether the role suited the participant

An internal facilitator was whether the role suited the participant. The participants felt the role suited them when 1) they had experienced the consequences of hand eczema, 2) they had strong communication skills, 3) the role fit with their other tasks, and 4) they had previous experience in a coaching-role. A comment of one of the participants was [Table 1, Quote 9]: ‘*What made it easier was that I followed other courses previously, for instance ergonomy training, and first aid, and then you already know what is to be expected from you as a coach.’* In addition, participants with experience as a coach reported to feel more confidence in guiding their colleagues in performing desired behaviour. This facilitator was also reported by a participant in an executive position [Table 1, Quote 10]: *‘That’s because I am in a position where it is perhaps easier for me to address people than for an analyst in the laboratory. That might be the case.’* In cases where the role did not fit the participant, this was experienced as a barrier. Several reasons were mentioned by the participants for why the role did not suit them: 1) the role model did not want to control other people, 2) the role model found it difficult to correct people, and 3) the role model had no experience in a coaching role. Of these factors, the lack of experience in a coaching role was mentioned most often. The manager illustrated why the role of role model was difficult for a young employee [Table 1, Quote 11]: ‘*J. just arrived, she had just finished her studies and she has just started to work here, and she was also in the working group… I think she was trying to find her own way. She does not have the experience yet to address people or to arrange these kind of things.’* Another participant mentioned that it was difficult performing in the role of a coach when they lacked supervisory experience.

#### Attitude towards the role

This factor was only mentioned as a facilitator. Role models reported that their attitude positively influenced their role when they 1) were motivated to perform in their role 2) had fun in being a role model [Table 1, Quote 12] and 3) took their tasks seriously.

### External factors

#### Support or resistance from colleagues and supervisors

Colleagues and supervisors could influence the functioning of the role models. On the one hand they could be supportive, which the role models experienced as facilitating, for instance when they wanted to arrange something for the project [Table 1, Quote 13]: *‘Well, the manager was also a member of the working group. That makes a difference when you wish to arrange things. Yes, that made it easier.’* On the other hand, a negative attitude towards the role model by department employees was mentioned as a barrier for performing their role. A manager explained that when one worker at his department was suddenly titled as a ‘coach’, this was not always accepted by his colleagues because they did not understand why they were not chosen [Table 1, Quote 14].

#### Contact between the role model and his/her colleagues and peers

To be able to perform their tasks, the role models indicated that having contact with their colleagues was of great importance. It was seen as a facilitator when the behaviour of their colleagues was well visible, as this gave them the possibility to address them when they performed unhealthy behaviour in relation to the prevention of hand eczema. On the other hand, not having contact with colleagues was deemed to be a barrier [Table 1, Quote 15]. Another participant explained that because of work pressure and lack of time it was not always possible to keep an eye on the behaviour of colleagues, something that was experienced as a barrier [Table 1, Quote 16]*.* When colleagues did not pick up or continue the encouraged healthy behaviour, this was experienced as a barrier to role models [Table 1, Quote 17]*.* When there were two or more role models within a department, and there was insufficient communication between them, this was experienced as a barrier for performing the role.

#### Risk perception

An external facilitator was the risk perception within the department of the role model. In departments where there was a colleague suffering from hand eczema, the problem was recognized by colleagues. One participant noticed how hand eczema among her colleague increased the awareness at the department [Table 1, Quote 18]; *‘We could have used that one colleague as an example, because it was very clearly visible and then you notice that people think: “Well, that’s not something I want, so I will do of course my best to avoid it.”’* Many role models mentioned that a low risk perception on hand eczema at the department was a barrier for performing their role. Several participants mentioned that their colleagues experienced no risk because they were not familiar with the problem [Table 1, Quote 19].

#### Number of role models

Two or more role models within a department, was described as a facilitator because they could support each other. A participant explained [Table 1, Quote 20]: *‘It was sometimes really nice to be with the two of us, because when one of us was silent, the other one could come up with other nice arguments.’* Taking charge of too many colleagues as a role model was experienced as a barrier.

#### Availability of material

The availability of study related material like flyers and leaflets about hand eczema, was experienced as a facilitator for performing the role. A participant explained that the role models used flyers to make their colleagues aware of the impact of hand eczema [Table 1, Quote 21].

#### Education

One participant explained that the educational session within his department contributed to the fact that his colleagues took his tasks more seriously afterwards.

#### When the role suited the department

Participants mentioned that it was a facilitator when the role suited the department where they worked. This was the case when employees were attainable in case of a small department for example, when there was a culture of speaking up and/or a dynamic atmosphere and when the workers of the department were used to paying attention to new projects [Table 1, Quote 22].

#### Priority of hand eczema

Several role models explained that the prevention of hand eczema was not relevant for the department because it was not a major issue at that moment [Table 1, Quote 23]. *O*ther priorities within the department, were also viewed as a barrier.

#### Low risk for hand eczema at the department

Another barrier for performing the role was having a low risk for developing hand eczema at work. At departments where people did not have to complete ‘dirty’ tasks or where they did not work with patients, employees had to clean their hands less compared to other departments. Therefore, role models did not always have the opportunity to give a good example for their colleagues.

## Discussion

The implementation strategy used in this study included role models who were intended to stimulate their colleagues to adhere to recommendations aiming to reduce hand eczema, and to pay attention to their colleagues’ risk behaviour. This study aimed to qualitatively explore what the role models perceived to be their main role and tasks, and to identify possible factors that hampered or facilitated their role.

### Main tasks of the role models

Creating awareness about ‘hand eczema’, the transferring of knowledge, and serving as a good example were perceived as important role model tasks. This is partly consistent with Ploeg et al. [4], who reported that dissemination of information by education and mentoring was one of the ways in which role models could influence the diffusion of best practice guidelines. In addition, coordinating the study happened to be one of the main tasks for participants in an executive function. It was not a goal of our study to investigate differences in tasks perceived by employees in a non supervisory position versus a supervisory one, but our findings suggest that differences may exist: role models in an executive function also felt responsible for coordinating tasks within the study, while participants without supervisory tasks often stuck with the tasks they received from role model training. However, it is also possible that managers who performed the role of role model would have taken care of coordinating tasks anyway, as it is often part of their job.

Previous research states that healthcare workers often do not think of themselves as role models and that they underestimate the impact of their behaviour on the behaviour of those they interact with [19]. We found that our participants considered giving a good example one of their main tasks. This finding suggests that a compact education session had an impact on the participants and that they were aware of the fact that their own behaviour might positively influence the behaviour of their colleagues. Remarkably, more participants with no executive function mentioned this task than role models with supervisory tasks. Previous research shows that role models, especially those in an executive function, should be aware of the influence of their behaviour on others, because medical students, for example, mentioned that they copy the behaviour of their superiors [13].

### Barriers and facilitators

The internal themes showed that the manner in which role models were selected, has room for improvement. Our participants were selected by their supervisor, based on their expected influence on colleagues and their motivation. Several participants mentioned that they experienced it as a barrier that they had to stay enthusiastic about a topic, that they would not mark as ‘highly important’ by themselves. Others mentioned that the role did not fit them, because they did not feel comfortable correcting the behaviour of their colleagues. Thus, it seems to be difficult for managers to assess whether someone is actually motivated and capable to perform a particular role. We suggest, for future interventions, that a ‘job application’ is written up prior to the study, in which interested participants can apply for the role of role model.

Two major facilitating factors for the role models were when the role fit in with other tasks, and when the role model had previous experience in a coaching role. These results suggest that it might be easier to act as a role model when someone is familiar with coaching-tasks, which is often the case when someone has supervisory tasks in their daily work. Furthermore, a manager explained that there was resistance among the employees when someone was suddenly entitled to be a coach. In previous studies where only the more senior health care workers acted as a role model [8, 19, 24] these findings were not reported. Also in our study, no managers reported the barrier of not being able to guide their colleagues to alter their behaviour. Therefore, in future role modeling interventions it might be better to choose workers with experience in coaching-tasks or with an executive function to act as a role model. When role models lack experience with coaching-tasks, a more comprehensive training about coaching needs to be incorporated.

An important external factor was the amount of support the role models received from their supervisor and/or their peers. When the role model felt supported by their colleagues or supervisor, this was experienced as a facilitator. A lack of social support and resistance towards the role model among colleagues was mentioned as a barrier. While the topic of ‘dealing with resistance’ is briefly discussed during role model training, we advise to pay more attention to resistance during the study. In addition, most barriers were experienced from the moment a role model had actually started to perform his/her role. Therefore it seems appropriate to plan meetings for the role models in which problems like lack of social support can be discussed with the other role models. We hypothesize that these peermeetings during the intervention might have the potential to increase their role confidence.

A low risk perception at the department was perceived as a barrier. Although we did not investigate whether the risk perception of the healthcare workers was correct, other studies indicate that there are many misperceptions among hospital staff regarding hand eczema prevention measures [25]. Colleagues of the role models in our study might have underestimated the risk of developing hand eczema. Educating role models and their colleagues about the occupational risk factors for developing hand eczema and the possibilities for prevention is therefore important. This was confirmed by our finding that several role models stated that they lacked sufficient knowledge about (the prevention of) hand eczema. Although an educational session was part of our implementation strategy, increasing the number of sessions in which the principles of prevention and the consequences of hand eczema are emphasized and refreshed once in a while should be considered. Furthermore, training the role models on peer-to-peer coaching could possibly contribute to a better performance.

### Strengths and limitations

Although the experiences of role models have been studied in wet work settings [5], this is the first study in a healthcare setting that examined facilitators and barriers experienced by the role models themselves. The explorative qualitative design provides more insight into the experiences of role models than a quantitative study could do. Thereby, important indepth information was obtained about how role models experienced their role and what they perceived to be barriers and facilitators which can be used for future implementation studies. Another strength is that our study population was heterogeneous: our data reflected perspectives from role models in different functions, positions and gender.

A limitation of our study is that participants might have been more enthusiastic about their role and the Hands4U study than role models who did not answer the request to participate in the interviews. The fact that we do not have data of the total group of role models may have biased our results. Furthermore, bias due to social desirability may have occurred because all interviews were conducted by the principal researcher of the Hands4U study. Participants were familiar with the researcher and her role. As a result, participants might not have mentioned all external barriers (like lack of support from Hands4U). Nevertheless, there was an open atmosphere during all interviews and participants were given the opportunity to add topics at the end. Bias may have occurred because not all interviews were double read and marked. Therefore, some quotes about tasks, barriers and/or facilitators might have been missed. However, as an inter-subjective agreement was tested on six interviews and quickly achieved, we assume that in the other interviews no quotes were missed.

## Conclusion

There were several factors that hampered and/or enhanced role models to perform their tasks. Important theme’s were: whether the role suited the participant, affinity with the topic of ‘hand eczema’ and support versus resistance from colleagues. Future interventions should take into account that role models should have experience with coaching-tasks or as a supervisor. As we consider the one hour and a half educational session as rather limited, a more comprehensive educational session could possibly contribute to a better performance of the role models. Furthermore, role models should have affinity with the study topic and be motivated to perform their role. During the intervention phase, role models can be better supported by ‘dealing with resistance’ training or by increasing their knowledge on the study topic.
